# Synthesis of highly fluorescent green carbon quantum dots from Prunus armeniaca for the determination of lisinopril in human plasma

**DOI:** 10.1038/s41598-025-17535-8

**Published:** 2025-09-12

**Authors:** Baher I. Salman

**Affiliations:** Pharmaceutical Analytical Chemistry Department, Faculty of Pharmacy, Al- Azhar University, Assiut branch, Assiut, 71524 Egypt

**Keywords:** N@CQDs, Content uniformity test, Lisinopril, Human plasma, Fluorescence, Analytical chemistry, Sustainability

## Abstract

The environmentally friendly synthesis of carbon quantum dots (CQDs) using natural precursors is garnering considerable interest. Nonetheless, questions may arise concerning the properties and efficacy of CQDs obtained from identical natural sources when originating from different geographical or botanical contexts. In this study, juice extracted from Prunus armeniaca (apricots) was employed as a natural precursor to produce N@CQDs using a one-step microwave-assisted technique. The photoluminescent properties of the synthesized nitrogen-doped carbon quantum dots (N@CQDs) demonstrated significant superiority over previously reported quantum dots, achieved through a more environmentally friendly and cost-effective synthesis method. The quantum yield of these N@CQDs reached an impressive 37.1%, while their nanoscale dimensions (approximately 2.6 nm) and chemical composition remained consistent with those reported in earlier studies. Subsequently, these nanoprobes were evaluated for their ability to detect the antihypertensive drug lisinopril (LIS). The results indicated that the N@CQDs exhibited both selectivity and sensitivity for LIS detection in bulk powder and biological plasma samples, within a concentration range of 5.0–150.0 ng mL^− 1^. The lower limit of quantitation was determined to be 2.2 ng mL^− 1^. The presence of LIS resulted in a significant reduction in the luminescence intensity of the synthesized green and stable N@CQDs at 502 nm (with an excitation wavelength of 455 nm). Additionally, the reusability and stability of the proposed CQDs for LIS analysis were thoroughly validated.

## Introduction

Hypertension is a prevalent condition, particularly among the elderly population. Often, it presents without noticeable symptoms, which may lead individuals to be unaware of its presence. Approximately 1.28 billion individuals between the ages of 30 and 79 globally are affected by hypertension, with a significant majority (about two-thirds) residing in low- and middle-income nations^1–3^. Furthermore, it is estimated that 46% of adults suffering from hypertension remain unaware of their condition. Over the last two decades, the development and advancement of nanomaterials have experienced significant growth^4–7^. These materials have demonstrated a wide range of applications and are increasingly preferred across various scientific disciplines due to their sustainable nature, biocompatibility, and minimal environmental impact. Carbon quantum dots (CQDs) represent a prominent category within this class of materials, characterized by their distinctive optical and electronic properties^8,9^. The nano-probes demonstrated unique properties, notably their high solubility in water and exceptional stability^10,11^. Carbon quantum dots (CQDs) serve as highly fluorescent probes that can be tailored to interact with specific substrates or analytes, resulting in changes in their fluorescent emissions^12–15^. Research into the synthesis of these zero-dimensional carbon-based nanomaterials has explored various economical, sustainable, and renewable sources. Among these, fruits stand out as excellent precursors for CQD synthesis due to their abundance of functional groups, which are present in the form of proteins, carbohydrates, vitamins, and other bioactive compounds^16–18^. The fluorescent probes are designed to interact with target substrates or analytes, resulting in variations in the fluorescent output^12,19–21^. The extensive applications associated with carbon quantum dots (CQDs) have attracted significant scholarly attention in this area of research^22,23^. CQDs have shown significant potential for both qualitative and quantitative detection of inorganic salts, owing to their functionalization with ligands that selectively bind to various inorganic substrates, as well as organic ones^24,25^. Additionally, numerous techniques have been proposed for synthesizing carbon quantum dots (CQDs) through bottom-up methods like hydrothermal, microwave-assisted, solvothermal, and electrochemical processes, or through top-down methods such as laser ablation, arc discharge, and high-energy ball milling^26,27^. The method of synthesis plays a crucial role in determining the performance and characteristics of the produced quantum dots. It can affect their size and composition, quantum yield, stability, and fluorescent properties^28,29^.

**Fig. 1:**
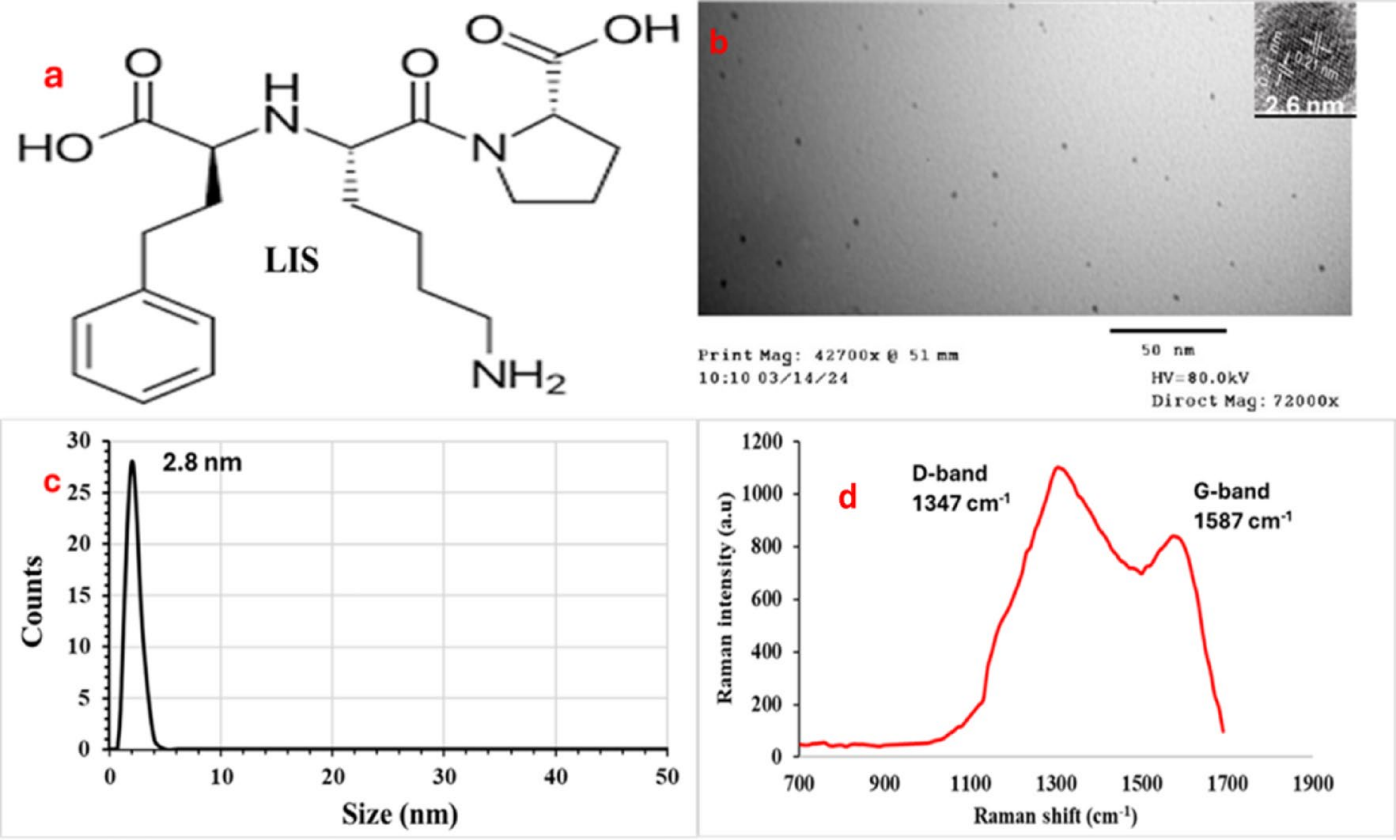
(**a**) Chemical structure of LIS, (**b**) TEM image, (**c**) Zetasizer, and (**d**) Raman spectroscopy for N@CQDs.

Lisinopril (LIS, Fig. 1a) is an angiotensin-converting enzyme (ACE) inhibitor that has received approval from the FDA for the treatment of hypertension and heart failure. LIS is frequently prescribed after a myocardial infarction, significantly contributing to the reduction of risks associated with subsequent strokes, heart attacks, and renal issues^30^.

Different techniques have been observed for the quantification of LIS, such as spectrofluorimetric^31,32^spectrophotometry^33–38^chromatography^34,37,39–41^and electrochemical^42^. The fluorimetric technique is an ideal choice for analyzing different products due to its selectivity, sensitivity, and cost-effectiveness compared to other methods^43,44^. As depicted in Fig. 1a, the LIS’s chemical structure features multiple functional groups, such as -NH_2_, -COOH, -NH, and C = O, enabling efficient binding with N@CQDs. Therefore, efficient, precise, biocompatible, and cost-effective detection methods are essential for analyzing pharmaceutical products (LIS), and these attributes are inherent to the approach proposed in this research. The proposed method was rapid, eco-friendly, and had a high quantum yield compared with the reported method^45^. The reported CQDs were processed from apricot using hydrothermal techniques using an autoclave with a long time for preparation^45–47^. The fluorescence intensity of the N@CQDs was quenched after the addition of different concentrations of LIS in a short period.

The main objective of the proposed research is to establish a novel and practical microwave-assisted synthetic method to produce innovative carbon quantum dots (N@CQDs) derived from apricots, a sustainable resource. This study focuses on the characteristics of the synthesized nitrogen-doped (N@CQDs) and their quantum yield (QY). Subsequently, the N@CQDs were explored as nano-biosensors for the detection of LIS across different matrices, and their performance was assessed against existing methodologies, particularly in terms of efficacy and environmental sustainability. The ecological implications of the entire process were evaluated using various greenness metrics, and the potential of this approach to improve the detection and quantification of LIS in both biological samples and tablet formulations was critically examined.

## Methodology part

### Chemicals and reagents

A high-purity (99.70%) powder LIS and Sinopril dosage form were obtained from GLOPAL NAPI Pharmaceuticals Company, Egypt. *Prunus armeniaca* fruits were acquired from the local market in Egypt. LIS standard solution (100 µg mL^− 1^) was prepared by dissolving ten mg of authentic powder (LIS) in a 100-ml volumetric flask with distilled water. Plasma samples were obtained from the Egyptian blood bank (Cairo, Egypt).

### Application of N@CQDs to formulated dosage form and content uniformity

Ten tablets of Sinopril^®^ (10 mg) were precisely weighed. Each tablet was mechanically pulverized to create a fine powder, corresponding to 10 mg of LIS, and then dissolved in 50 mL of distilled water using sonication for 5 min. The solution was subsequently diluted to a final volume of 100 mL with more distilled water. Following this, filtration was performed, and the supernatant was gathered for further analysis as outlined in the relevant subsection.

### Investigation of LIS in human plasma samples

Plasma samples were maintained at a temperature of -20 degrees Celsius before undergoing analysis. A combination of 0.5 mL of drug-free plasma and different concentrations of LIS was prepared. To aid in protein precipitation, 0.5 mL of methanol was added. The mixture was subsequently diluted to a final volume of 10 mL with double-distilled water and centrifuged at 5000 rpm for 20 min. Ultimately, 1.0 mL of the supernatant was collected for the analysis of LIS.

### Instrumentation

The green quantum dots synthesis was performed using an MFMI-100 A Microwave (900 W, Jinan, China). A Shimadzu Spectrophotometer (double beam, Japan) was used for spectrophotometric analysis. JEM High-Resolution Transmission Electron Microscope (HRTEM) (JEM-1400 Plus, Tokyo) was utilized to study N@CQDs. Fluorescence results were measured using FS2, with a 1.0 cm quartz sample cell, and an ozone-free xenon light source (150 W) was used during the experiment, which was obtained from Sinco (Korea). Examination of FT-IR spectra of N@CQDs was carried out using an FT-08 spectrometer (Wakendorf, Germany). Metrohm pH meter 780 from Metrohm (Herisau, Switzerland). Philips X-ray diffractometer (model PW3040/00) was used for scanning the powder X-ray from Philips Innovation lab (Eindhoven, Netherlands).

### Synthesis and characterization of the developed green N@CQDs

The green synthesis of the N@CQDs was obtained as reported in salman et al.^48^. The apricot fruit was first processed in a mixer to extract the juice after the pit was removed. A 50 mL aliquot of the juice was then placed in a conical flask and exposed to microwave radiation at 900 watts for 5 min, resulting in a brown solution. Following this, the solution underwent filtration, was sonicated for 20 min, and centrifuged at 4000 rpm for 10 min, before being filtered again through a 0.45 μm cellulose membrane. The final solution was prepared for subsequent analysis and characterization. Key characterization parameters for the synthesized N@CDQs included size and shape determination, which were performed using TEM imaging and DLS. The surface chemistry was analyzed using FTIR techniques. The optical properties of the synthesized CQDs were evaluated through UV-Vis and photoluminescence spectroscopy. Additionally, energy-dispersive X-ray (EDX) and powder X-ray diffraction (PXRD) spectra were employed to assess the composition and structure of the N@CQDs.

### Reusability study of quantum dots as fluorescent probes

A recycling test was carried out to determine the reusability of N@CQDs, focusing on the quantification of LIS (50 ng mL^- 1^) medication over ten iterations within a ten-week timeframe. At the end of each iteration, the quantum dots were separated using centrifugation, thoroughly cleaned with ultra-pure water and ethanol, and dried for 30 min at 50 °C to ensure their reusability. The quantum dots were then kept at 4 °C for future utilization.

### Stability study of LIS in human plasma using the green carbon quantum dots

The stability study of LIS in human plasma using the N@CQDs method was performed at three quality control levels: low (LQC 10.0 ng mL^- 1^), medium (MQC 50.0 ng mL^- 1^), and high (HQC 100.0 ng mL^- 1^).

### Stability study of N@CQDs under different conditions

The stability of N@CQDs plays a significant role in determining their viability for multiple applications. This research focused on evaluating the stability of N@CQDs under various conditions. (1) They were exposed to a temperature range of 25 to 90 degrees. A total of 1.0 mL of N@CQDs was added to 10-mL volumetric flasks and brought to volume with distilled water. These flasks were then placed in a digital water bath at different temperatures ranging from 25 to 90 °C for 30 min. After cooling, fluorescence measurements were taken at 502 nm. (2) UV examination was carried out as follows: 1.0 mL of N@CQDs was added to 10-mL volumetric flasks and diluted to the mark with distilled water. The flasks were placed under a UV lamp at 360 nm, and the fluorescence was assessed at various times. (3) To assess pH stability, solutions with pH values ranging from 2 to 12 were formulated by adjusting the concentrations of 0.1 M NaOH and 0.1 M HCl solutions, followed by dilution. Then, 0.5 mL of the diluted N@CQDs solution was added to each of the solutions with the indicated pH levels. The pH values were measured using a pH meter, and the fluorescence spectra were recorded at 502 nm.

### Spectrofluorimetric procedure

The synthesized N@CQDs were utilized to evaluate their sensitivity and responsiveness to LIS across different matrices. The interaction between the N@CQDs and LIS was analyzed at varying concentrations to determine their effectiveness as reliable probes for the detection and quantification of this significant pharmaceutical compound. To create the calibration graph, a 1.0 mL solution of N@CQDs dispersion (concentration of 0.25 mg mL^− 1^) was combined with 1.0 mL of Britton-Robinson (BR) buffer at pH 7.6. Following this, 1.0 mL of LIS standard solution was introduced within a concentration range of 5.0 to 150.0 ng mL^− 1^. The mixture was thoroughly agitated, and the total volume was adjusted to 10 mL with ultra-pure water. After a 10-minute incubation, the fluorescence intensity was recorded at 502 nm, using an excitation wavelength of 455 nm.

## Results and discussion

*Prunus armeniaca (*Apricot*)* was classified among the top dietary sources of polyphenols and can be classified as a natural functional food. Their consumption is linked to various health benefits, such as hepatoprotection, anti-inflammatory properties, and antihypertensive effects, among others. The phenolic content in apricots can differ based on the variety, maturity, and cultivation methods used. However, it is well-documented that apricots are abundant in flavonoids, particularly flavanols and flavonols, as well as non-flavonoid phenolics, notably hydroxycinnamic derivatives^49–52^. Apricot primarily contains flavonols in the form of quercetin and kaempferol glucosides, with quercetin-3-O-rutinoside (rutin) being the most significant component^49–52^. Flavanols are predominantly found as either (+)-catechin or (−)-epicatechin, with the dominant type varying by apricot variety. Additionally, various dimeric flavanol forms can reach notable concentrations^49–52^. Among the hydroxycinnamic acids, chlorogenic acid is frequently identified as the principal compound. Other compounds such as neochlorogenic acid, p-coumaric acid, caffeic acid, and gallic acid may also be present in various apricot cultivars. Besides, Fatty acids were reported as oleic, palmitic, and linoleic acids^53^. Some oils are also present as sitosterol and stigmasterol^53^. Furthermore, small amounts of other phenolic compounds, including resveratrol, can be detected. Therefore, apricots were chosen as the ideal green source for carbon quantum dots.

### Characteristic features of N@CQDs

The surface morphology of N@CQDs was analyzed using Transmission Electron Microscopy (TEM) as shown in Fig. 1b. The TEM analysis revealed an average particle size of 2.6 ± 0.27 nm, with a lattice spacing of 0.21 nm. However, the selected area electron diffraction (SAED) pattern, as shown in (Supplementary Materials; Fig. S1), demonstrates that the N@CQDs are amorphous, which is also supported by the XRD spectrum in Fig. S2. The PXRD analysis of N@CQDs shows a significant diffraction peak around 26.40°, which aligns with the [002] pattern (Fig. S2). This finding indicates that the synthesized N@CQDs exhibit an amorphous structure, likely due to the closely packed carbon atoms with alkyl branches and a notable number of oxygen-containing groups on their surface^48,54^.

**Fig. 2:**
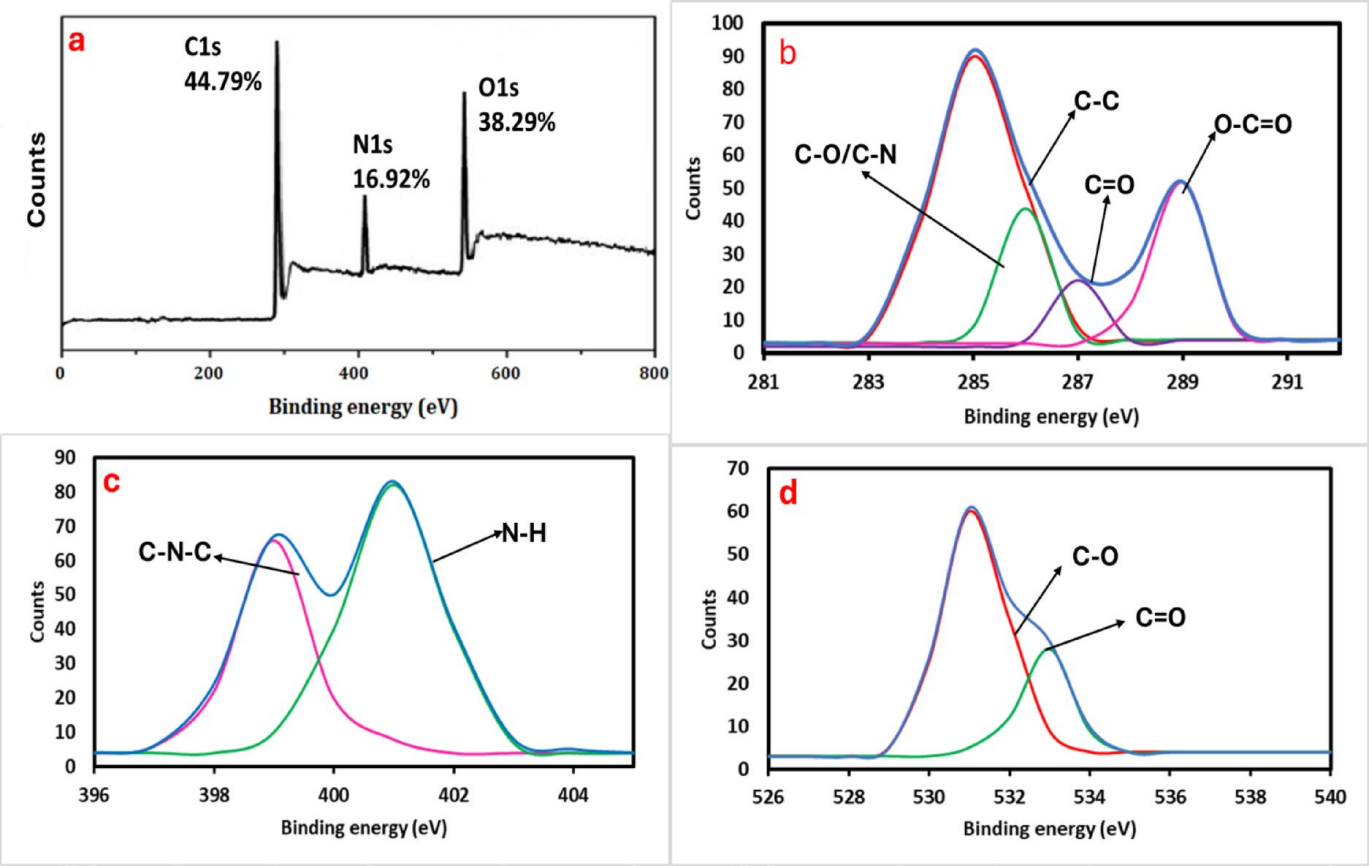
Elemental analysis of N@CQDs; (**a**) XPS spectrum, (**b**) C 1s spectra, (**c**) N 1spectra and (**d**) O 1s spectra.

Furthermore, the particle size distribution of N@CQDs was assessed using a zeta sizer through Dynamic Light Scattering (DLS), resulting in a measurement of 2.8 ± 0.29 nm, as illustrated in Fig. 1c, along with a monodispersity value of 0.14. Additionally, the structural characteristics of N@CQDs were confirmed through Raman spectroscopy. The Raman spectrum presented in Fig. 1d highlights two significant peaks at 1347 and 1587 cm^− 1^, which correspond to the D and G bands of N@CQDs. A comprehensive elemental analysis conducted through EDX confirmed the presence of carbon (C), nitrogen (N), and oxygen (O) elements, as shown in Fig. S3. The findings indicate that the composition of carbon dots consists mainly of C (44.79%), O (38.29%), and N (16.92%). The X-ray photoelectron spectroscopy (XPS) analysis elucidates the elemental composition and bonding features of carbon dots. The survey spectrum reveals the presence of three primary elements, each identified by its unique binding energies: carbon at 287.16 eV, nitrogen at 403.24 eV, and oxygen at 535.19 eV, confirming the successful integration of these elements into the carbon dot framework (Fig. 2a). The C 1s spectra (Fig. 2b) display four distinct peaks: sp2 carbon and C–C bonds at 284.98 eV, C–N/C–O bonds at 285.87 eV, C = O groups at 287.31 eV, and (O-C = O) at 289.04 eV. This indicates a sophisticated carbon structure with various functional groups. The N 1s spectrum (Fig. 2c) reveals two peaks at 399.87 eV and 401.57 eV, representing C-N-C and N-H groups, respectively. The O 1s spectrum shows two different oxygen environments: one peak at 533.15 eV corresponding to C = O bonds and another at 531.81 eV associated with C–O bonds (Fig. 2d). In addition, the surface morphology of the N@CQDs was analyzed using FTIR spectroscopy, as shown in Fig. S4. The FTIR spectrum reveals characteristic peaks corresponding to the (-NH, -OH) –CH groups, which are detected at 3430 and 2920 cm^− 1^, respectively. Moreover, the peaks at 1640, 1420, and 1210 cm^− 1^ are linked to the –C = O, –CN, and -C-O-C functional groups.

**Fig. 3:**
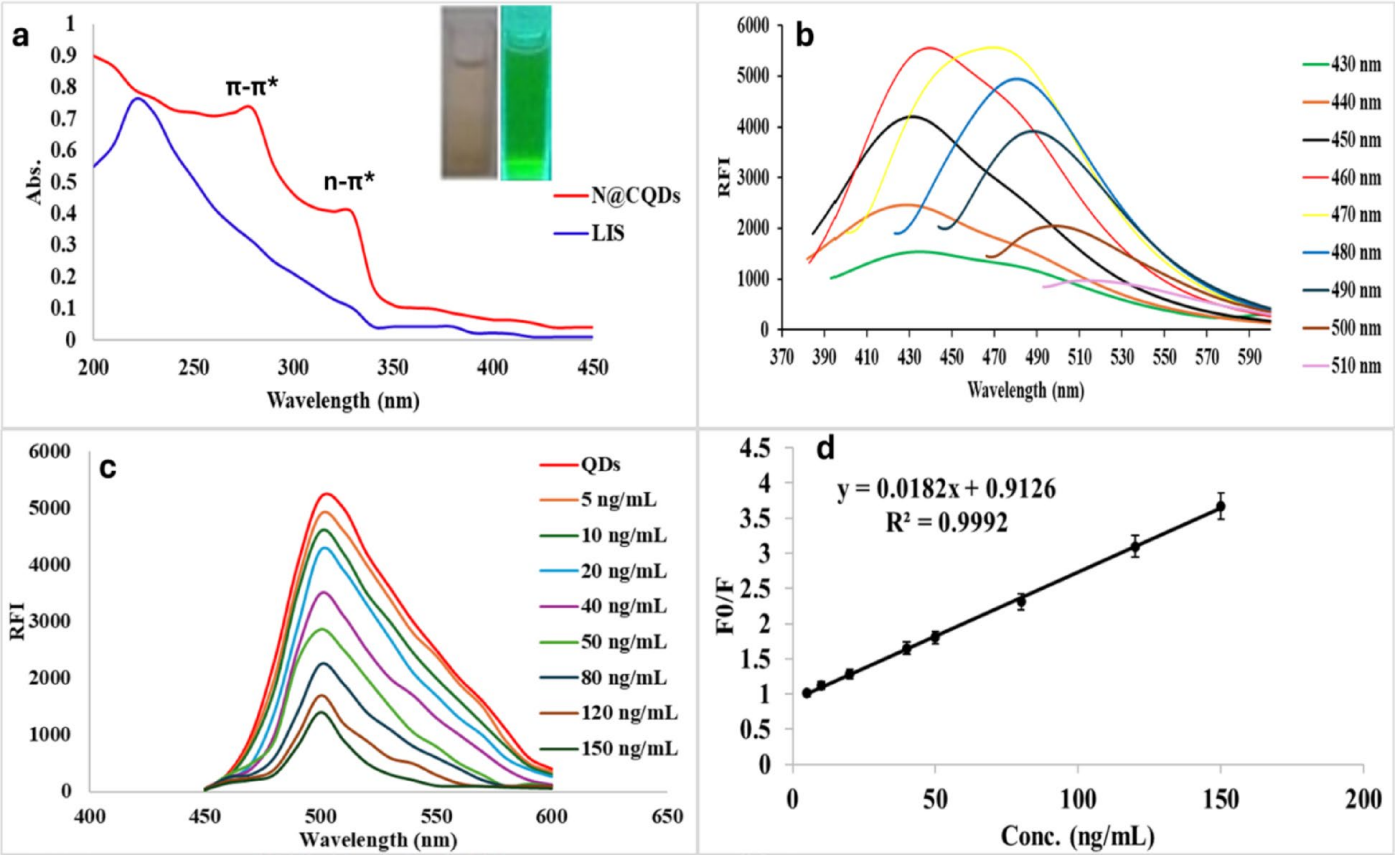
(**a**) Optical activity of N@CQDs using UV, (**b**) Excitation dependent emission, (**c**) Reaction of N@CQDs with LIS and (**d**) Linear range of LIS for reaction with N@CQDs.

The optical examination of N@CQDs identified two significant peaks at 285 nm and 330 nm, as shown in Fig. 3a^55^. These peaks are indicative of the carbon structure and the electronic transitions of the functional groups^55^. The luminescence spectra of N@CQ dots were analyzed by modifying the excitation parameters. With increased excitation, a red shift was observed in the emission spectra of N@CQ dots, transitioning from 430 nm to 510 nm. This shift was accompanied by a decrease in RFI, which confirms the excitation-dependent nature of the emission from the carbon dots^56^. Figure 3b.

Furthermore, the reaction of LIS with N@CQDs was conducted at 502 nm (Fig. 3c) in the linear range 50.0–150.0 ng mL^− 1^ (Fig. 3d).

The quantum yield of the green synthesized QDs was determined using the subsequent formula^55^. The following equation was used, where Qst is the fluorescent QY of the standard (QY rhodamine B = 0.31), *η* represents the refractive index of water as solvent, and *I* is the fluorescence intensity.

$$\:{\varvec{Q}}_{\varvec{X}}={\varvec{Q}}_{\varvec{s}\varvec{t}}\:.\:\frac{{\varvec{I}}_{\varvec{X}}}{{\varvec{I}}_{\varvec{s}\varvec{t}}}.\:\frac{{\varvec{A}}_{\varvec{s}\varvec{t}}}{{\varvec{A}}_{\varvec{X}}}.\:\frac{{\varvec{\eta\:}}^{2}}{{\varvec{\eta\:}}^{2}}$$

The (QY) of N@CQDs was calculated to be 37.1%.

Evaluating the fluorescence stability of N@CQDs is crucial for assessing their applicability in real-world scenarios. This research examined the behavior of N@CQDs under various conditions, including exposure to a broad temperature spectrum (25 to 90 degrees), a defined UV irradiation intensity for 7 h, and pH stability. The fluorescence intensity remained stable throughout all time points during the heating test, with no decline noted in comparison to the initial fluorescence intensity illustrated in Fig. S5a. To tackle this specific issue, an extensive study was conducted to evaluate the photo-stability performance of the N@CQDs. The N@CQDs were subjected to UV light at 360 nm while in ambient air for different durations. The results, shown in Fig. S5b, clearly indicate that there is no significant alteration in the fluorescence intensity of the N@CQDs during the testing period with 30-minute intervals. Notably, even after a lengthy exposure of 7 h, the N@CQDs maintain 95% of their original fluorescence intensity. Furthermore, the resistance to pH was evaluated across a range from 2 to 12. The results indicate that N@CQDs maintained stability between pH 2 and 11, underscoring the resilience of QDs (Fig. S5c) even though they underwent slight quenching at pH 2 and moderate quenching at pH 12.

### The optimization process of the N@CQDs approach

**Fig. 4:**
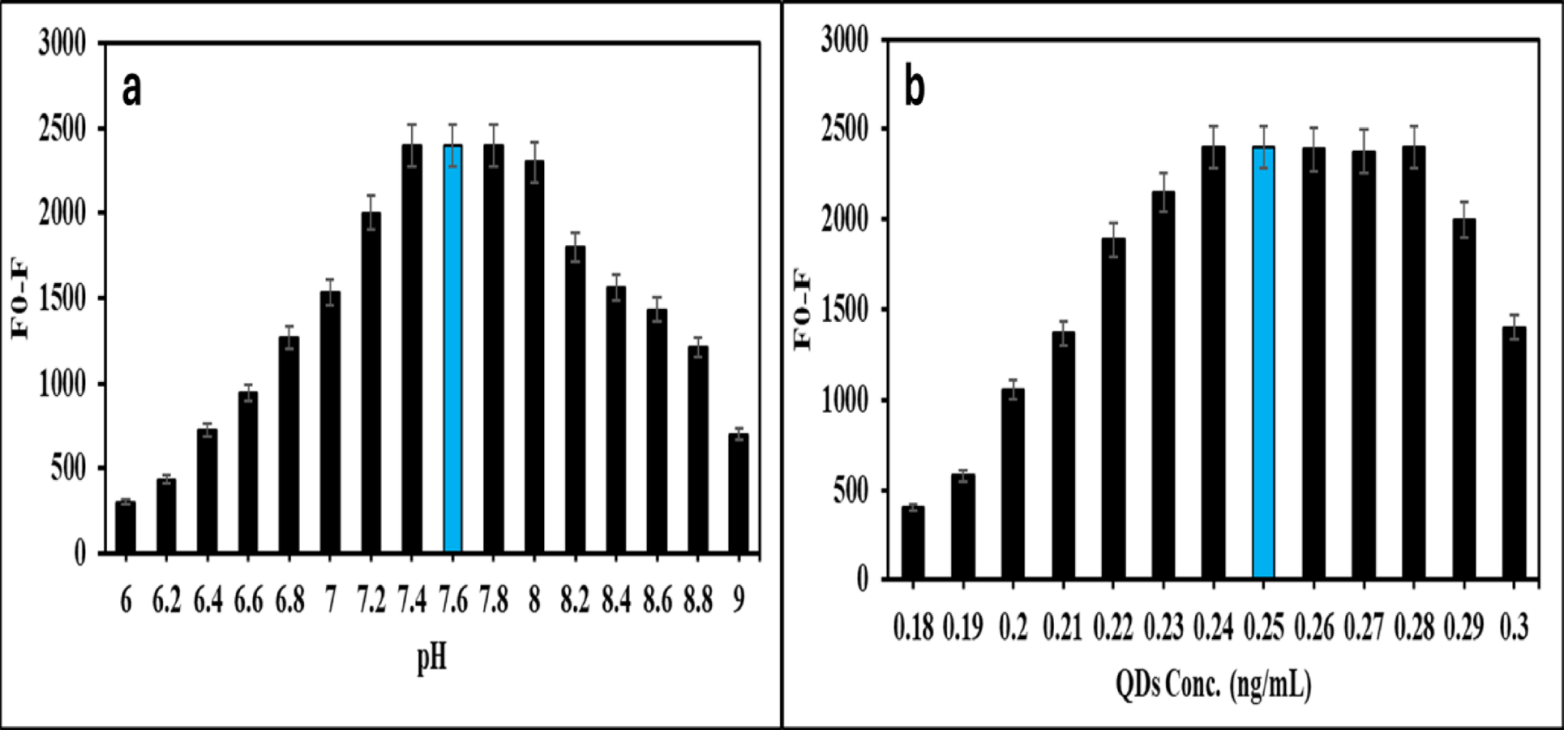
Effect of pH (**a**) and effect of the concentration of N@CQDs (**b**) for the reaction with LIS (50 ng mL^− 1^).

The factors affecting the sensitivity of the method were carefully examined and adjusted to boost the reactivity of LIS towards N@CQDs. Various variables included the interaction time with LIS, the volume and pH of the buffer used, and the concentration of N@CQDs. The buffer’s pH was analyzed within the range of 6.0 to 9.0 during the interaction of LIS with the fluorescent N@CQDs. The peak fluorescent signal was achieved at pH 7.6 (Fig. 4a) using 1.0 ± 0.25 mL of BR buffer. A study was performed to investigate the impact of N@CQDs concentration on its fluorescence interaction with LIS. Various concentrations of N@CQDs, specifically between 0.18 and 0.3 mg mL^− 1^, were evaluated. The results indicate that the optimal relative fluorescence intensity (RFI) occurred at a concentration of 0.25 mg mL^− 1^, as shown in Fig. 4b. The study concentrated on the effects of interaction time, which was analyzed over up to 40 min (Fig. S6). The findings indicated that the ideal duration for completing the interaction was 10 min. Any time spent beyond this threshold was found to be ineffective and did not influence the RFI (Fig. S6). Additionally, the parameters for synthesis (synthesis duration, microwave power, and precursor volume) for the creation of green N@CQDs were meticulously optimized. Various microwave power levels (600, 700, 900, 1000, and 1200 watts) were assessed for the synthesis of N@CQDs over different time intervals. It was determined that 900 watts for 5 min yielded a high quantum yield. Moreover, different volumes of apricot juice were examined (ranging from 10 to 100 mL) for N@CQDs optimization. It was observed that 50 mL produces a high quantum yield, as shown in Table S1**.**

### Reaction mechanism of the proposed method

The quenching effect of LIS when reacting with N@CQDs (Fig. 3c) was facilitated by multiple mechanisms, such as static and dynamic quenching, the internal filtering effect (IFE), and fluorescence resonance energy transfer (FRET)^57,58^. Figure 3a presents data that reveals a distinct absorption spectrum of LIS at 215 nm, which does not interfere with the absorbance spectrum of N@CQDs at 285 and 330 nm. This finding suggests that a FRET mechanism is not taking place during the quenching process. Furthermore, there is no spectral overlap between the absorption spectrum and the emission spectrum of N@CQDs at 502 nm, especially in the internal filter effect^57,58^which inhibits the inner filter effect. Therefore, the Stern-Volmer equation was used to distinguish between static and dynamic quenching using the following equation:$${\raise0.7ex\hbox{${{\text{F}}_{{\text{0}}} }$} \!\mathord{\left/ {\vphantom {{{\text{F}}_{{\text{0}}} } {\text{F}}}}\right.\kern-\nulldelimiterspace} \!\lower0.7ex\hbox{${\text{F}}$}}{\text{ = 1 + Ksv}}\,{\text{[Q]}}$$.

The linearity shown in Fig. 3d indicates that a dynamic quenching mechanism is present. The interaction between LIS and excited N@CQDs enhances energy and electron transfer, which accounts for the quenching of quantum dot luminescence^23,59^. To provide additional evidence for the dynamic quenching mechanism, experiments were carried out on the reaction between N@CQDs and LIS at various temperatures. The results revealed that an increase in temperature corresponded with a rise in the Stern-Volmer constant, thereby supporting the dynamic quenching hypothesis^60^. Fig. S7.

Furthermore, to evaluate the electrical charges present on the surface of N@CQDs and their stability in colloidal form, a zeta potential analysis was conducted before and after the reaction. The results of the zeta potential measurements for N@CQDs are illustrated in Fig. S8. The zeta potential was determined to be − 12.64 mV, -20.51 mV, and − 5.73 mV for N@CQDs, LIS, and the complex (LIS-N@CQDs), respectively, indicating the complexation between LIS and N@CQDs. In addition, FTIR spectroscopy was performed for N@CQDs in the presence of LIS, as shown in Fig. S9. It was observed that diminishing the intensity of FTIR peaks was diminished due to the binding with LIS.

To explore the quenching behavior of the N@CQDs, fluorescent lifetime measurements were taken both with and without the quencher molecule (Fig. S10). The average fluorescent lifetimes were 7.30 ns and 8.47 ns in the presence and absence of LIS, respectively. These findings indicate that the presence of LIS caused a significant change in the lifetime of N@CQDs. This result suggests a dynamic quenching mechanism.

### The N@CQDs method validations

The fluorescence technique for analyzing the interaction between N@CQDs and LIS has been validated following ICH guidelines and has received bioanalytical validation in compliance with the standards established by the United States Food and Drug Administration (US-FDA)^61,62^.

Figure 3c represents the gradual addition of LIS to the quantum dots, which causes the quenching effect measured at 502 nm (excitation 455 nm). The calibration graph was established by plotting the relative fluorescence intensity (RFI) against various concentrations of LIS utilizing the Stern-Volmer equation:

$${\raise0.7ex\hbox{${{\text{F0}}}$} \!\mathord{\left/ {\vphantom {{{\text{F0}}} {\text{F}}}}\right.\kern-\nulldelimiterspace} \!\lower0.7ex\hbox{${\text{F}}$}}{\text{ = 1 + Ksv}}\,{\text{[Q]}}$$

In the context of nitrogen-doped carbon quantum dots (N@CQDs), the fluorescence efficiencies are indicated as F0 in the absence of LIS and F in its presence. The Stern-Volmer constant, Ksv, serves to measure the quenching process, while the concentration of the quencher is [Q]. The calibration range was set between 5.0 and 150.0 ng mL^− 1^, as shown in Table 1. The limits of detection (LOD) and quantification (LOQ) were calculated based on the slope and standard deviation (σ) of the calibration curve. The LOQ was determined using the formula (10 σ/slope), while the LOD was calculated as (3.3 σ/slope). The resulting LOD of 0.73 ng mL^− 1^ and LOQ of 2.20 ng mL^− 1^ (Table 1) highlight the exceptional sensitivity of the analytical method proposed. To estimate the reliability of the N@CQDs method for quantification of LIS, five concentrations (10.0, 20.0, 50.0, 100.0, and 150.0 ng mL^− 1^) were analyzed within the calibration range. The recovery percentages varied between 100.36 ± 0.29% and 101.80 ± 0.36%, demonstrating the high accuracy of the N@CQDs method. Table 2.

**Table 1:** Quantification parameters for analysis of LIS using the proposed technique.

Parameter	Results
λ_ex_ (nm)	455
λ_em_(nm)	502
Concentration range (ng mL^− 1^)	5.0 –150.0
Determination coefficient (r^2^)	0.9992
Slope	0.0182
Intercept	0.912
SD the intercept (Sa)	0.004
LOD (ng mL^− 1^)	0.73
LOQ (ng mL^− 1^**)**	2.2

LOD: lower limit of detection, LOQ: lower limit of quantitation.

**Table 2:** Accuracy and precision studies for assay of LIS using the proposed approach.

Sample number	Actual concentration (ng mL^− 1^)	Found concentration (ng mL^− 1^)	% Recovery ± RSD^*^
1	10.0	10.15	101.50 ± 0.42
2	20.0	20.33	101.65 ± 0.58
3	50.0	50.18	100.36 ± 0.29
4	100.0	101.28	101.80 ± 0.36
5	150.0	151.25	100.83 ± 0.37
Intra-day precision	30.0	30.21	100.70 ± 0.27
	60.0	61.01	101.68 ± 0.48
	90.0	90.36	100. 40 ± 0.35
Inter-day precision	30.0	29.91	99.70 ± 0.64
	60.0	59.13	98.55 ± 0.89
	90.0	90.05	100.05 ± 0.41

^*^: Average of three determinations; RSD: Relative standard deviation.

To evaluate the precision of this method, both intra-day and inter-day variations were analyzed. This involved measuring three different concentrations of LIS at 30.0, 60.0, and 90.0 ng mL^− 1^, with three measurements taken on the same day and three separate days. The results confirmed the method’s exceptional precision (Table 2).

**Fig. 5:**
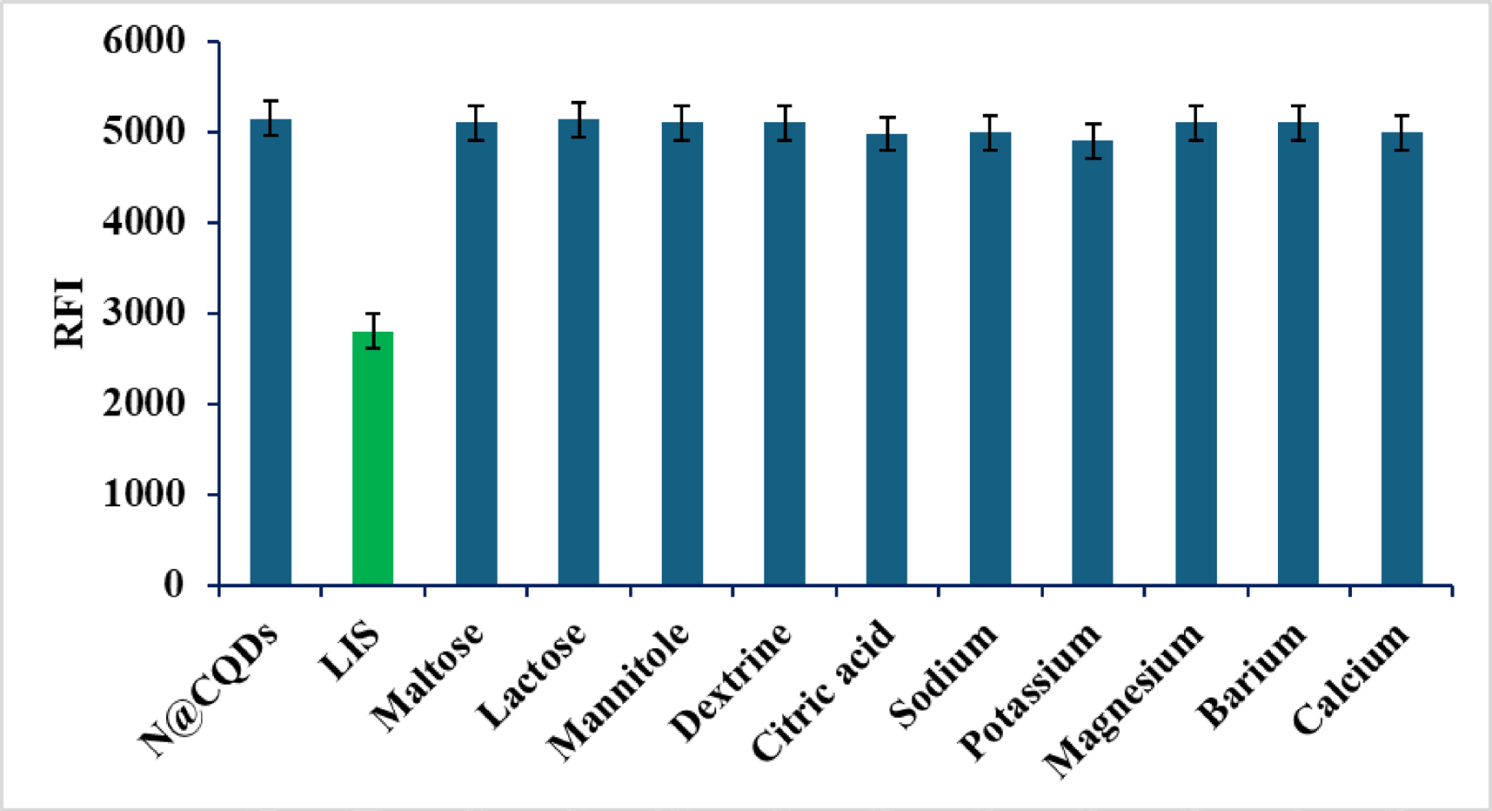
Selectivity study for analysis of LIS (50 ng mL^− 1^) in the presence of different interferences.

The selectivity of the N@CQDs method was conducted for analysis of LIS in the presence of interfering additives (0.5 µg mL^− 1^). The study investigated various interfering additives, such as citric acid, mannitol, dextrin, lactose, maltose, and different metal ions (magnesium, sodium, calcium, potassium, and barium), revealing the method’s strong selectivity, as these additives had little to no effect on the fluorescence intensity of N@CQDs (Fig. 5).

Additionally, bio-analytical validation and assessments of matrix effects were performed at three concentrations: 40.0, 80.0, and 100.0 ng mL^− 1^, all within the calibration range. As shown in Table 3, the relative standard deviation (RSD) values ranged from 1.76 to 2.26. The results indicated the absence of any interference of the matrix with a high percentage of recovery. Table 3.

**Table 3:** Matrix effect study of the N@CQDs method for analysis of LIS in human plasma.

Conc. (ng mL^− 1^)		Intra-day assay (*n* = 6)		Inter-day assay (*n* = 9)		
		Accuracy (%)		Precision (CV %)	Accuracy (%)	Precision (CV %)
40.0		98.12		2.30	98.00	2.26
80.0		97.15		1.81	96.88	1.90
100.0		98.29		1.97	97.95	1.76

The study also investigated the stability of LIS in human plasma across three quality control levels: low (LQC 10.0 ng mL^− 1^), medium (MQC 50.0 ng mL^− 1^), and high (HQC 100.0 ng mL^− 1^). The recovery rates, presented in Table 4, varied from 96.91 ± 1.73% to 98.13 ± 1.27%, demonstrating that LIS maintains excellent stability in human plasma.

**Table 4:** Stability study of LIS in human plasma using different parameters.

	LQC 10.0 ng mL^− 1^	MQC 50.0 ng mL^− 1^	HQC 100.0 ng mL^− 1^
Three Freeze–thaw cycle stability (-24 °C)	98.21 ± 1.10	98.01 ± 1.60	98.23 ± 2.44
Long-term stability (1 month at -24 °C)	97.46 ± 1.15	98.12 ± 1.73	97.51 ± 2.16
Short-term stability (12 h at -24 °C)	97.77 ± 1.42	97.50 ± 1.44	97.90 ± 1.38
Post-preparative stability (6 h at room temperature 25 °C)	98.13 ± 1.27	97.67 ± 1.18	96.91 ± 1.73
Post-preparative stability (12 h at room temperature 25 °C)	96.95 ± 1.94	97.15 ± 1.62	97.67 ± 2.06

Data presented as recovery (%) ± SD (*n* = 5).

An assessment of the robustness of the proposed study was conducted to determine whether slight alterations in the experimental parameters would influence the quenching of N@CQDs fluorescence intensity by the specified medication. The variations examined included the volume of N@CQDs, BR, and the buffer volume. The results indicated that these minor changes in experimental conditions did not impact the quenching of N@CQDs fluorescence intensity by LIS, as presented in **Table S2**.

### Investigation of N@CQDs reusability for estimation of LIS

The assessment of LIS (50.0 ng mL^[- [1^) depicted in Fig. S11 was conducted over five iterations to evaluate the reusability of carbon dots. The findings revealed that the RFI system consistently performed well during the recycling analysis, highlighting the impressive reusability of carbon dots over ten cycles. Thus, N@CQDs show great potential for extended utilization.

### Applications of N@CQDs to various matrices

To enhance the effectiveness and the applications of the proposed nanoprobes, different matrices were evaluated to quantify the levels of LIS. The LIS samples were spiked at different concentrations into plasma samples devoid of any medication. The results presented in Table 5 indicate a notable recovery rate between 96.97 ± 1.81% and 98.63 ± 1.23% for the analyzed drug from human plasma using the proposed method.

**Table 5:** Application of the green N@CQDs method for analysis of LIS in human plasma.

Added conc. (ng mL^− 1^)	Found (ng mL^− 1^)	% Recovery ± RSD
20.0	19.58	97.90 ± 2.92
40.0	38.79	96.97 ± 1.81
60.0	58.78	97.96 ± 2.00
100.0	98.63	98.63 ± 1.23
150.0	147.32	98.21 ± 1.75

* Mean of three determinations (*n* = 3).

The proposed technique successfully determined the LIS concentration in Sinopril^®^ 5 mg tablets, achieving a percentage recovery of 101.50 ± 0.28. These results underscore the method’s reliability and consistency.

The determination of LIS concentrations in low-dose tablets was conducted due to the high sensitivity and selectivity of the proposed method. An analysis of ten tablets was performed using this approach. The content uniformity of the tablets was evaluated according to the guidelines set by the United States Pharmacopeia^63,64^. The acceptance value (AV) was calculated and found to be below the maximum permissible acceptance value (L1) as recommended by the USP^23,65^as presented in Table 6.

**Table 6:** Quality control study for the estimation of LIS in using the quantum Dots method.

Tablet No.	% Recovery
	Sinopril^®^ (5.0 mg/tablet)
1	101.24
2	100.83
3	99.50
4	101.78
5	102.04
6	102.15
7	101.87
8	100.08
9	101.14
10	102.00
Mean	101.26
SD	1.05
Acceptance value (AV)*	2.52
Max. allowed AV (L1)*	15

* Acceptance value = 2.4 × SD.

### Assessing the proposed technique for analysis of LIS relative to some reported methods

Various methodologies have been previously documented for the determination of LIS utilizing different techniques. This study represents the first instance of LIS determination employing the innovative N@CQDs method. The proposed method was assessed against several previously established techniques for LIS determination (Table 7). Certain limitations may be observed in the other techniques that have been reported. For example, chromatographic methods, which are commonly used in laboratories today, have notable disadvantages when compared to spectrofluorimetric methods, particularly in terms of cost-effectiveness and sample processing capacity [33,34]. The financial investment required for the purchase and upkeep of chromatographic systems is significantly higher. Additionally, expenses related to columns, solvents, mobile phases, and other consumables can add up considerably, especially when dealing with a high volume of samples. In the realm of green chemistry, this approach presents an innovative strategy for the development and synthesis of chemicals, emphasizing the reduction of their harmful environmental impacts. It focuses on minimizing the use of organic solvents, reagents, and energy consumption in analytical processes. The innovative N@CQDs probes utilize water as an environmentally friendly solvent and eliminate the need for extraction, setting them apart from previously established methods. Additionally, as demonstrated, N@CQDs exhibit greater selectivity and improved sensitivity, as indicated by the lower limit of detection (LOD) in comparison to other reported techniques. Table 7.

**Table 7:** Comparative study of the proposed method against the published methods.

Technique	Linear range	LOD	Applications	References
The proposed method	5.0-150 ng mL^− 1^	0.73 ng mL^− 1^	Dosage form and human plasma	
Spectrophotometric	2.0–26.0 µg mL^− 1^ 25.0–300.0 µg mL^− 1^	0.038 µg mL^− 1^ 0.27 µg mL^− 1^	Dosage form	^33^
Spectrophotometric	2.0–20.0 µg mL^− 1^	0.30 µg mL^− 1^	Human plasma	^34^
Spectrophotometric	10.0–150.0 µg mL^− 1^	5.58 µg mL^− 1^	Dosage form	^36^
Spectrofluorimetric	0.05–1.0 µg mL^− 1^	20.0 ng mL^− 1^	Dosage form	^31^
Chromatographic	5.0-200.0 ng mL^− 1^	NA	Human plasma	^39^
Chromatographic	12.5–37.5 µg mL^− 1^	7.0 ng mL^− 1^	Dosage form	^40^

### Assessment of the environmental sustainability of the proposed method

Various tools have been unveiled for the assessment of the ecological repercussions of analytical methodologies such as the GAPI and AGREE methods^15,65^. Such assessments are vital for fulfilling sustainability aims by mitigating the environmental detriment caused by routine analytical chemistry activities^66,67^. Furthermore, the comprehensive ratings and color-coded representations of GAPI and AGREE pictograms indicate a reduction in red zones, thereby reflecting a more favorable ecological impact for the synthesized N@CQDs nanoprobes. Table 8.

**Table 8:**
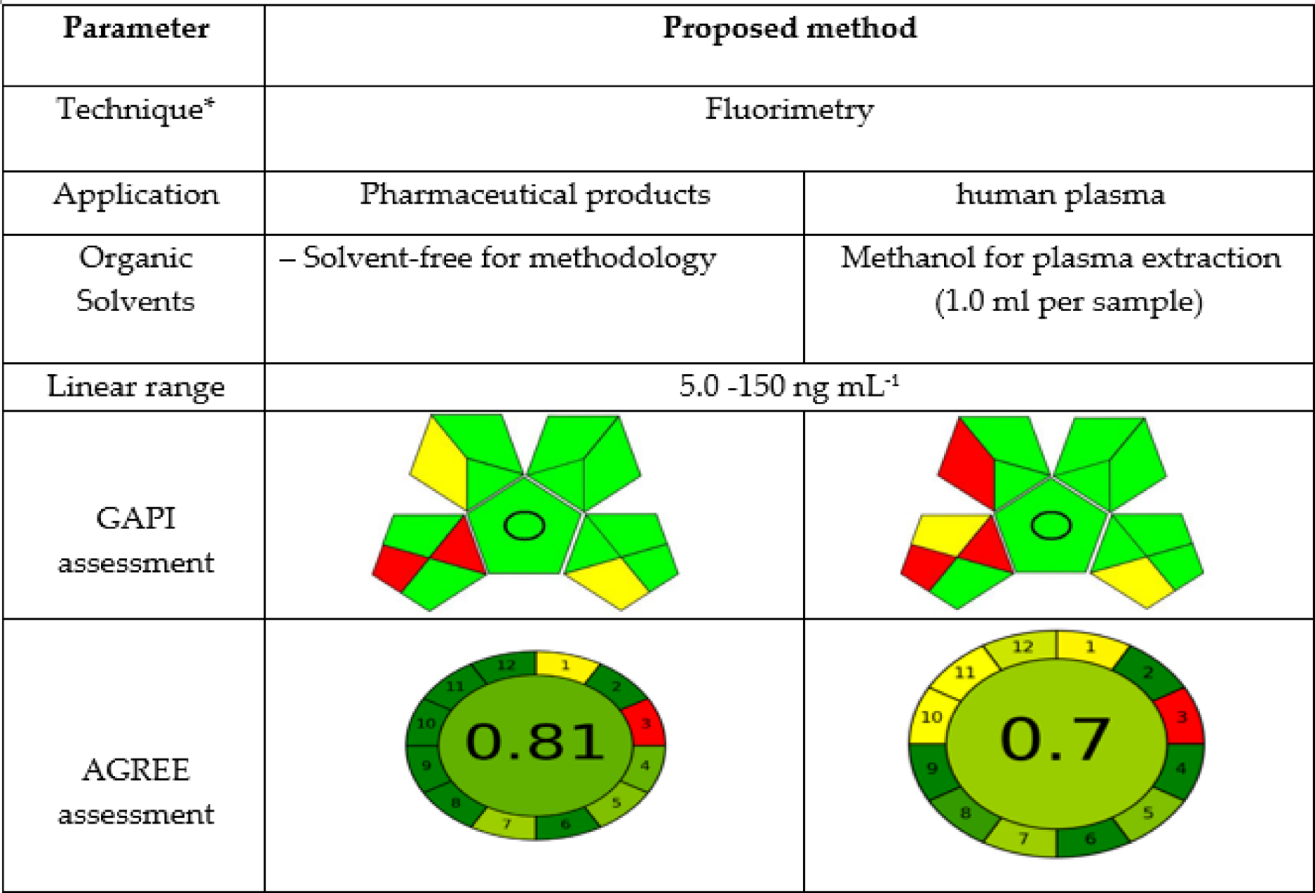
Greenness study for N@CQDs method for analysis of LIS.

## Conclusion

A new, simple, and cost-effective method for microwave-assisted synthesis has been developed and confirmed for the production of nitrogen-doped carbon quantum dots (N@CQDs). This cutting-edge technique yields highly fluorescent green N@CQDs with an impressive quantum yield of 37.17%. Additionally, the environmentally friendly nature of this research aligns perfectly with the tenets of green analytical chemistry. This approach proves to be more effective for the regular analysis of LIS in pharmaceutical formulations and human plasma. By considering expected future developments and innovations, technologies based on N@CQDs could significantly improve pharmaceutical analysis, public health, and sensor technology. The integration of green chemistry principles with cutting-edge nanotechnology offers considerable potential for addressing challenges related to analysis and resource management.

## Supplementary Information

Below is the link to the electronic supplementary material.

Supplementary Material 1

## Data Availability

The datasets generated and/or analyzed during the current study are available in this article and its supplementary materials).
