# Supplementary material for: Synthesis of highly fluorescent green carbon quantum dots from Prunus armeniaca for the determination of lisinopril in human plasma
Source: Sci Rep. 2025 Sep 12;15:32502. doi: 10.1038/s41598-025-17535-8 (PMC12432171; doi:10.1038/s41598-025-17535-8)
Supplement: Supplementary file 1 — Supplementary Material 1 [file 41598_2025_17535_MOESM1_ESM.docx]

**Synthesis of highly fluorescent green carbon quantum dots from Prunus armeniaca for the determination of lisinopril in human plasma**

Baher I. Salman ^1*^

*^1^ Pharmaceutical Analytical Chemistry Department, Faculty of Pharmacy, Al-Azhar University, Assiut branch, Assiut, 71524, Egypt,* [bahersalman@azhar.edu.eg](mailto:bahersalman@azhar.edu.eg)

*** Corresponding author;**  [bahersalman@azhar.edu.eg](mailto:bahersalman@azhar.edu.eg)


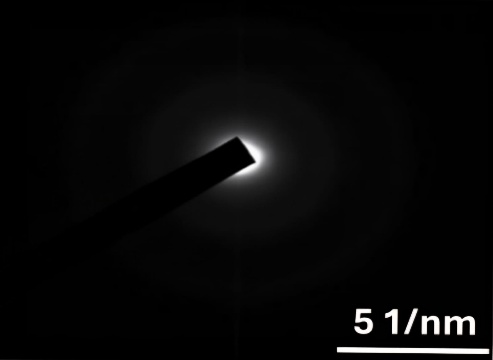


**Figure S1:** Selected area electron diffraction (SAED) pattern for N@CQDs.

**
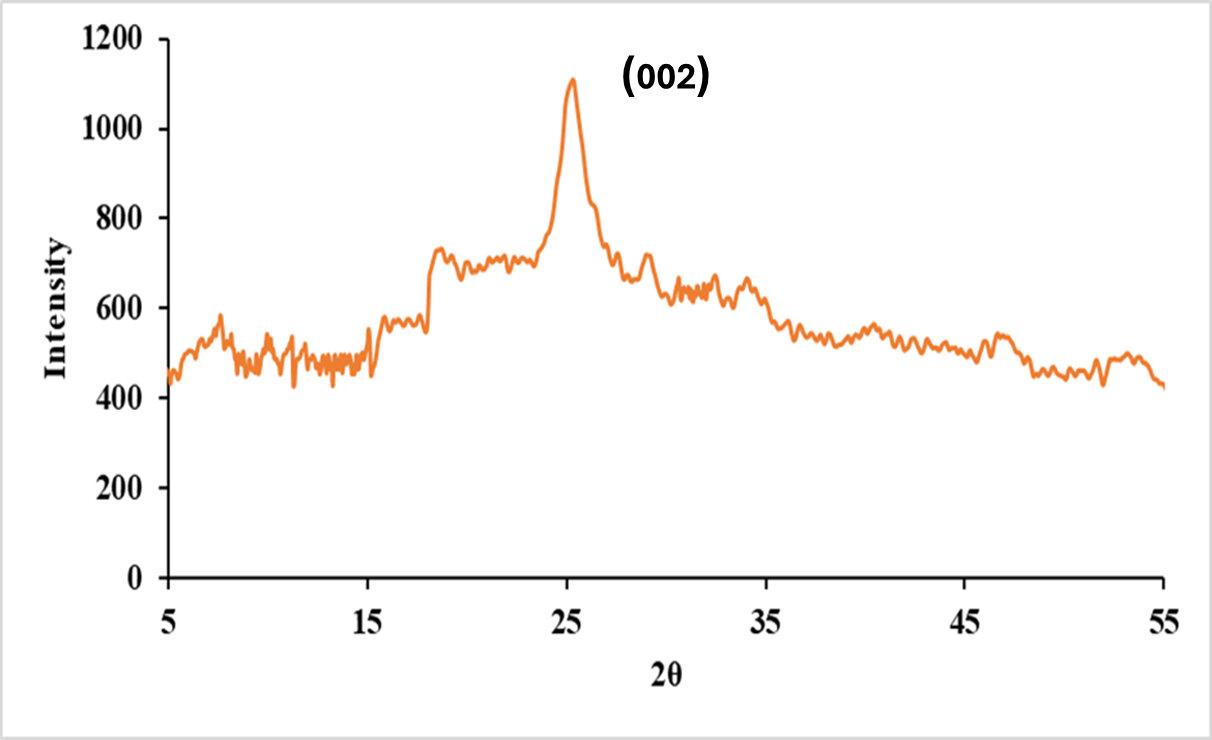
**

**Figure S2:** PXRD spectrum for the synthesized N@CQDs.


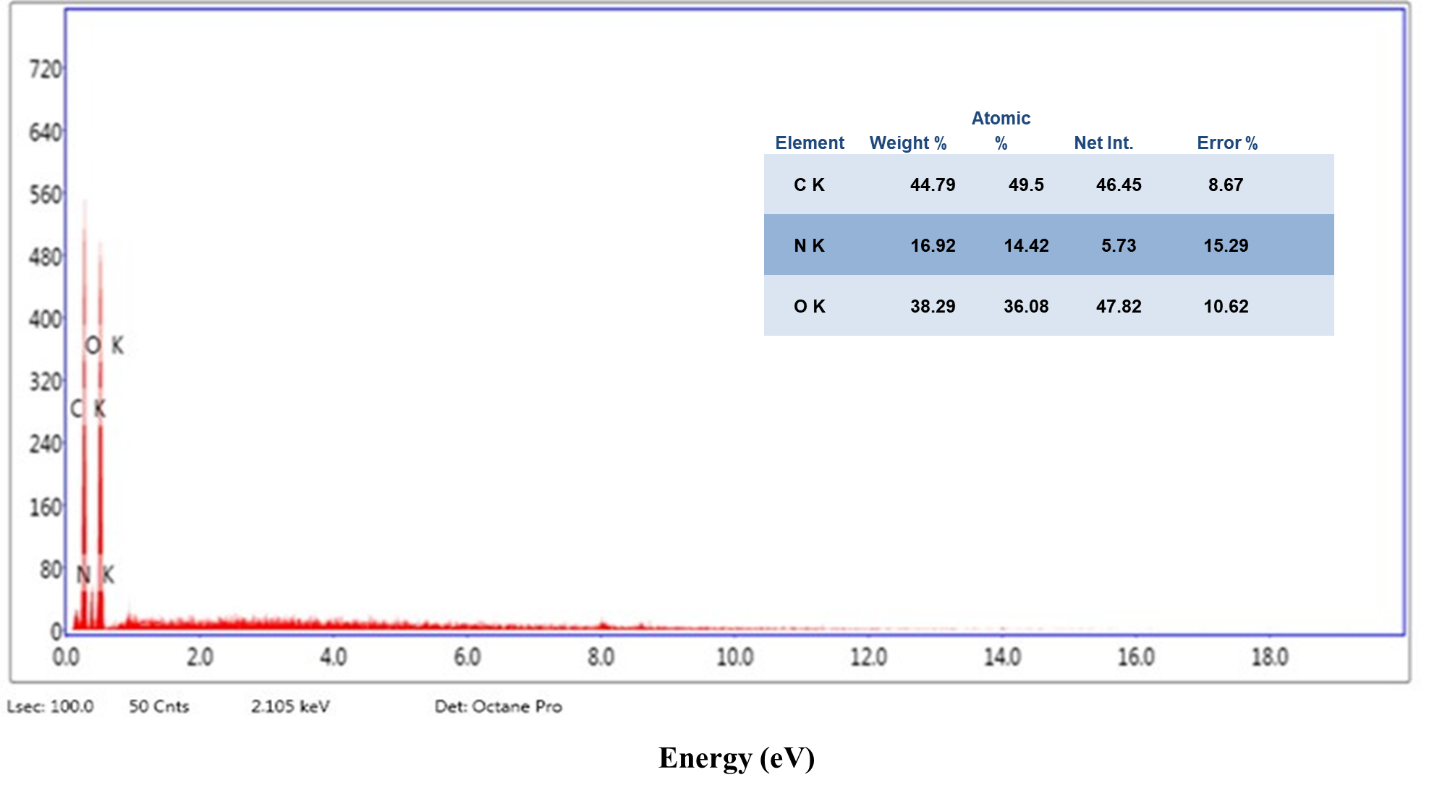


**Figure S3:** EDX spectrum for the naturally developed N@CQDs.

**
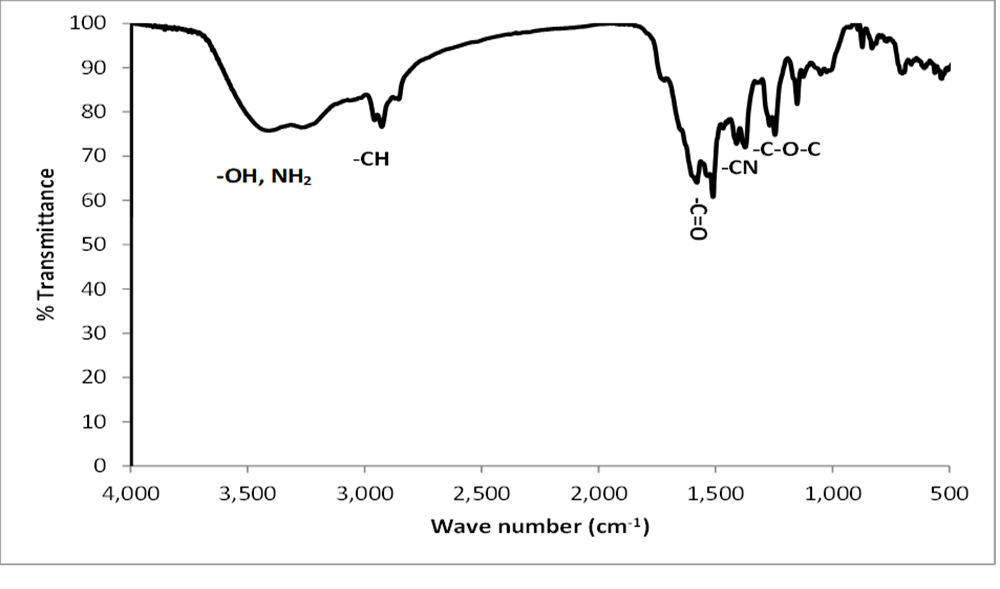
**

**Figure S4:** FTIR spectrum for N@CQDs.

**
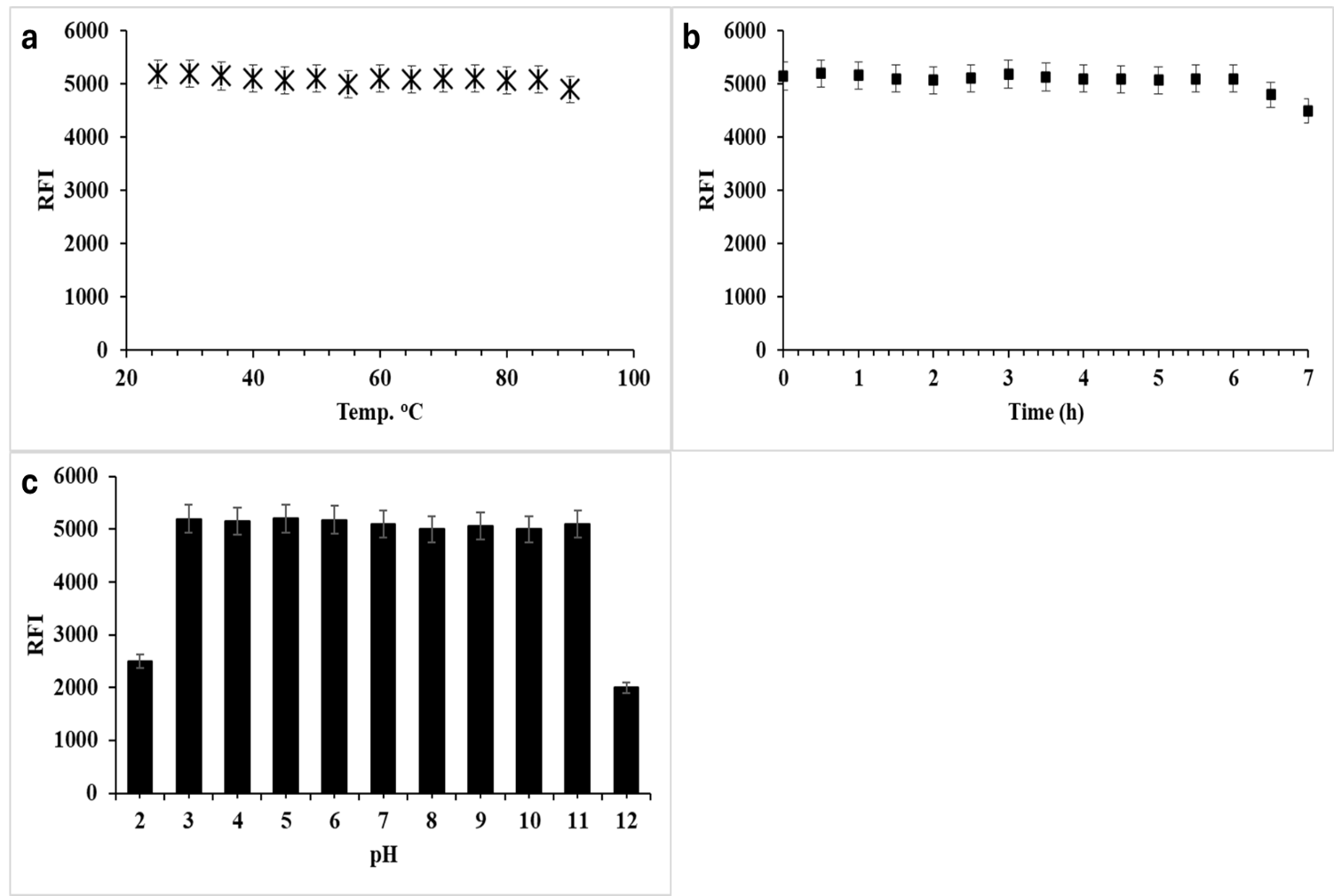
**

**Figure S5:** Stability study of N@CQDs under different conditions, **a)** Temperature, **b)** UV examination and **c)** pH stability.

**
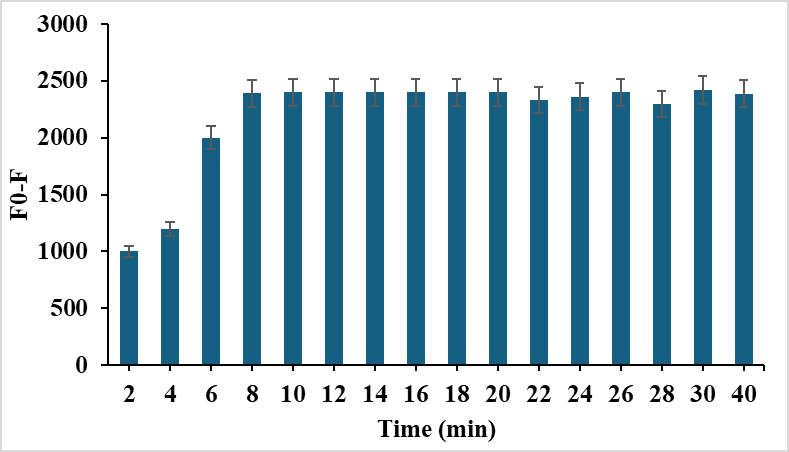
**

**Figure S6:** Influence of the reaction time on the RFI for reaction of LIS (50 ng mL^-1^) with N@CQDs.

**
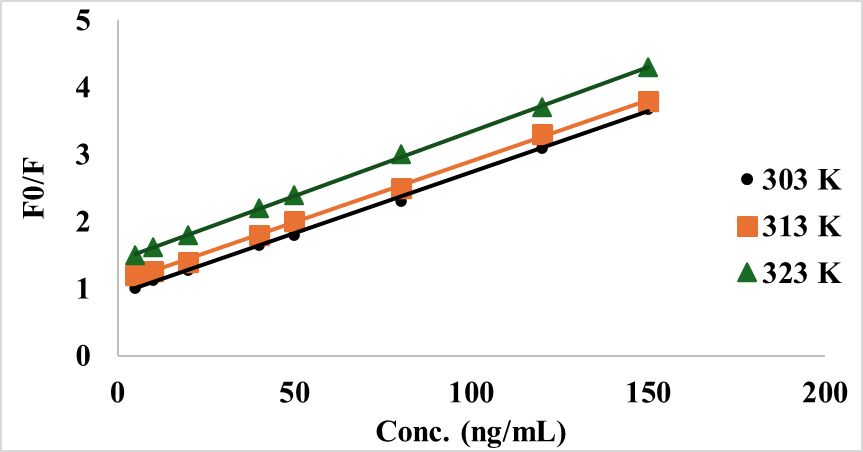
**

**Figure S7:** Effect of temperature on the stern-Volmer constant for reaction of LIS with N@CQDs.


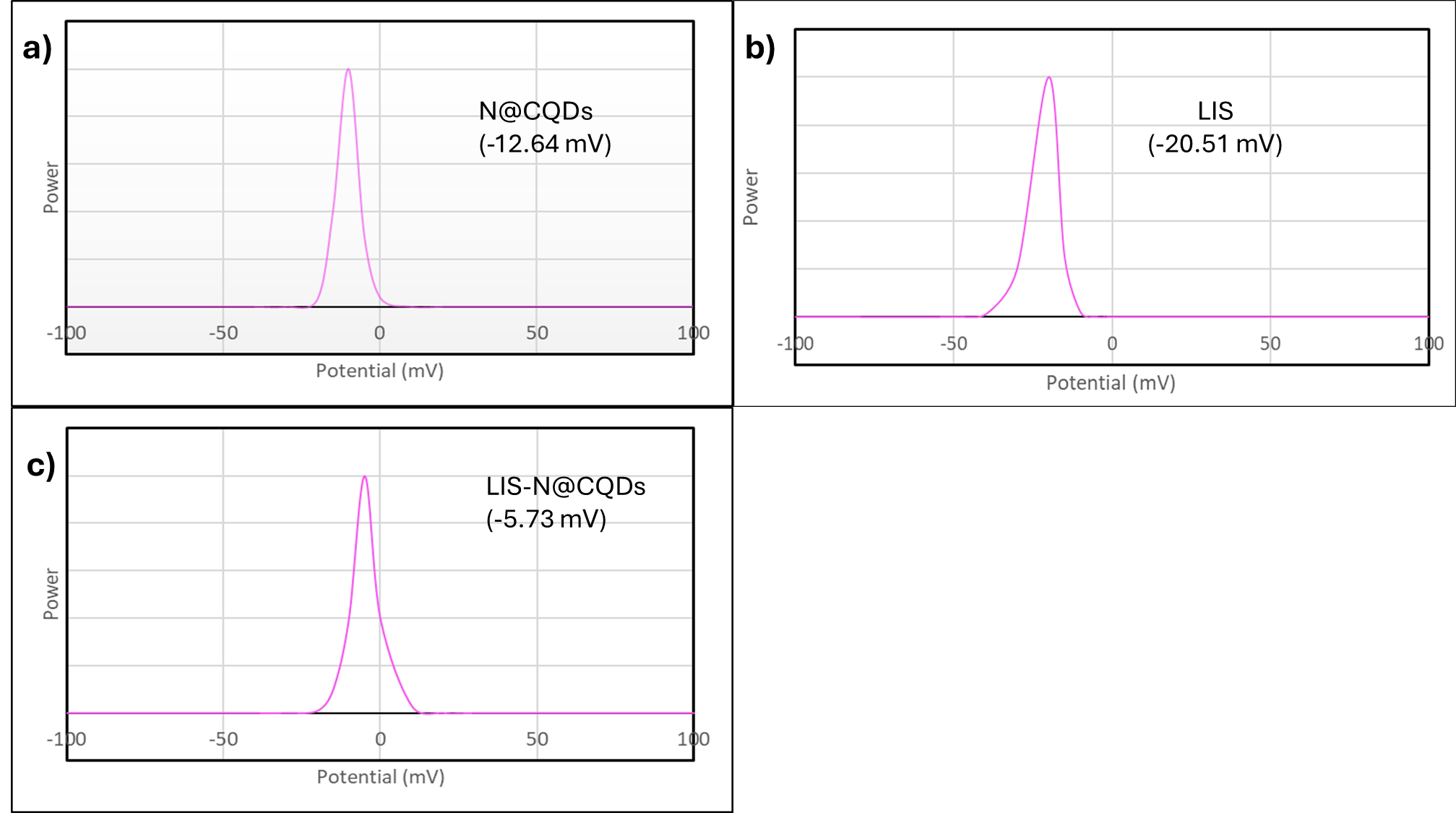


**Figure S8**: Zeta potential for the proposed method before and after the reaction


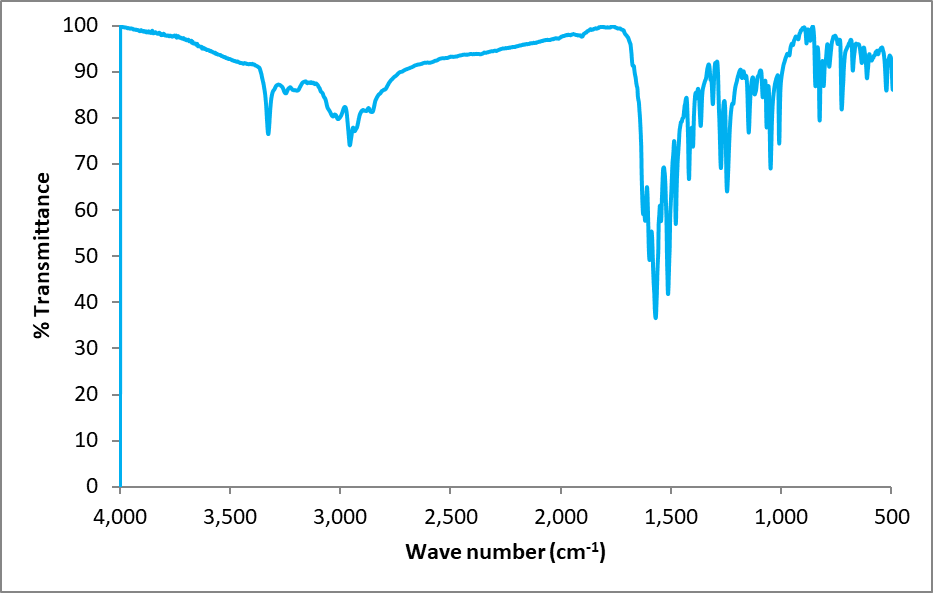


**Figure S9:** FTIR spectrum for N@CQDs in the presence of LIS.


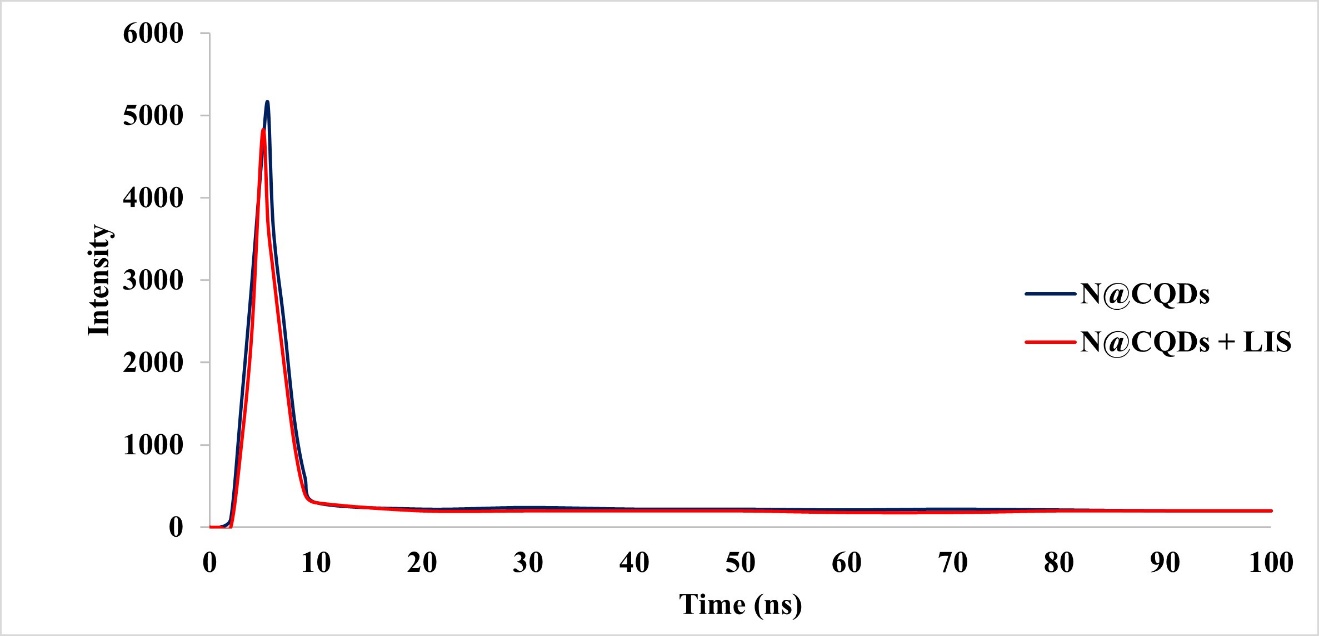


**Figure S10:** Time-resolved fluorescence spectroscopy for N@CQDs in the absence and the presence of LIS.


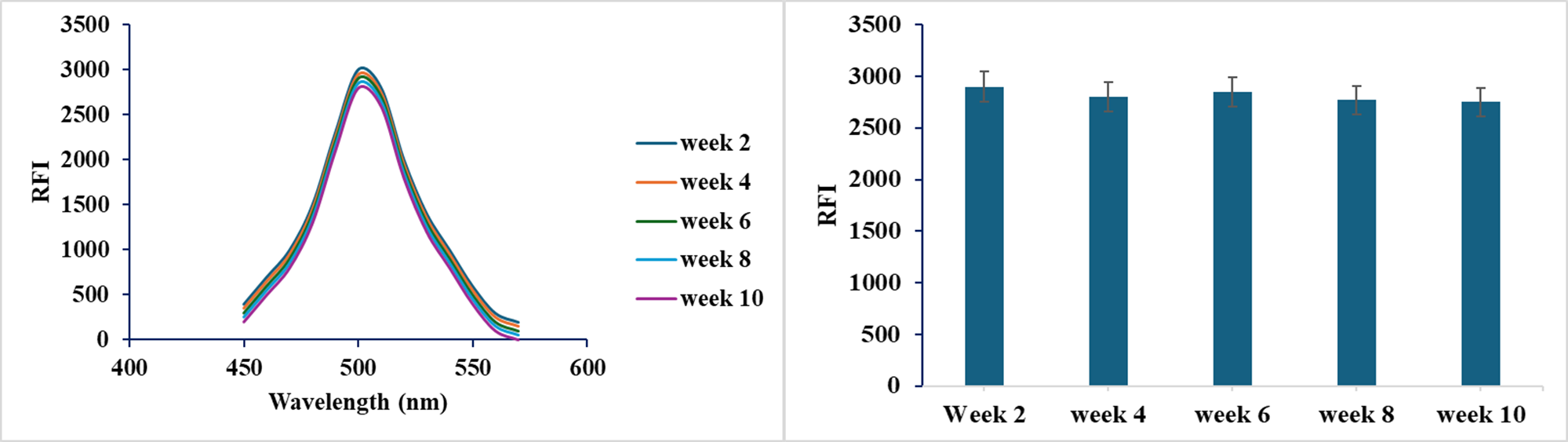


**Figure S11:** Reusability of the N@CQDs for determination of LIS (50 ng mL^-1^) 5 cycle.

**Table S1:** Synthesis parameters for optimize the N@CQDs

| **% QY+SD*** | |  |  |
| --- | --- | --- | --- |
| **Microwave power (Watt)** | | | |
| 25.3 ± 0.21 | | 600 | |
| 37.04 ± 0.18 | | 700 | |
| 37.09 ± 0.42 | | 900 | |
| 37.10 ± 0.17 | | 1000 | |
| 31.5 ± 0.30 | | 1200 | |
| **2- Synthesis time (min)** | | | |
| 20.47 ± 0.37 | | 2 | |
| 29.82 ± 0.24 | | 3 | |
| 37.09 ± 0.42 | | 4 | |
| 37.10 ± 0.25 | | 5 | |
| 37.06 ± 0.27 | | 6 | |
| 37.03 ± 0.41 | | 7 | |
| 31.32 ± 0.33 | | 8 | |
| 22.64 ± 0.47 | | 9 | |
| 18.93 ± 0.59 | | 10 | |
| **3- volume of precursor (mL)** | | | |
| 14.69 ± 0.40 | | 10 | |
| 28.22 ± 0.29 | | 20 | |
| 34.50 ± 0.19 | | 30 | |
| 37.11 ± 0.15 | | 40 | |
| 37.10 ± 0.26 | | 50 | |
| 37.09 ± 0.16 | | 60 | |
| 36.90 ± 0.37 | | 70 | |
| 36.98 ± 0.30 | | 80 | |
| 36.94 ± 0.41 | | 90 | |
| 37.05 ± 0.50 | | 100 | |

***:** Mean of six determinations.

**Table S2:** The robustness of the proposed N@CQDs method for the determination of LIS (100.0 ng mL^-1^).

|  | |  | **Variations** |
| --- | --- | --- | --- |
| **% Recovery ^a^ ± RSD** | |  |  |
| 101.52 ± 0.80 | |  | **Optimum condition** |
| **1- pH** | | | |
| 100.18 ± 0.82 | | 7.4 | |
| 100.54 ± 0.37 | | 7.7 | |
| **2- Volume of buffer (mL)** | | | |
| 100.88 ± 0.68 | | 0.75 | |
| 100.48 ± 0.27 | | 1.25 | |
| **3- N@CQDs concentration (mg mL^-1^)** | | | |
| 100.19 ± 0.85 | | 0.20 | |
| 100.68 ± 0.14 | | 0.30 | |
| **4- Reaction time (min)** | | | |
| 100.91 ± 0.49 | | 8 | |
| 100.14 ± 0.31 | | 12 | |

**^a^:** Mean of six determinations.
